# Reducing drug related deaths: a pre-implementation assessment of knowledge,barriers and enablers for naloxone distribution through general practice

**DOI:** 10.1186/1471-2296-15-12

**Published:** 2014-01-15

**Authors:** Catriona Matheson, Christiane Pflanz-Sinclair, Lorna Aucott, Philip Wilson, Richard Watson, Stephen Malloy, Elinor Dickie, Andrew McAuley

**Affiliations:** 1 Academic Primary Care, University of Aberdeen, Aberdeen, UK; 2 Division of Applied Health Sciences, University of Aberdeen, Aberdeen, UK; 3 RCGP (Scotland) Clinical Lead Addiction, Glasgow, UK; 4 Stephen Malloy Training and Consultancy, Glasgow (formerly Scottish Drugs Forum), Glasgow, UK; 5 Public Health Adviser (NHS Scotland), Thistle House, 91 Haymarket Terrace, Edinburgh, UK; 6 Public Health Adviser (NHS Scotland), Meridian Court, Glasgow, UK

## Abstract

**Background:**

The Scottish Naloxone Programme aims to reduce Scotland’s high number of drug-related deaths (DRDs) caused by opiate overdose. It is currently implemented through specialist drug services but General Practitioners (GPs) are likely to have contact with drug using patients and their families and are therefore in an ideal position to direct them to naloxone schemes, or provide it themselves. This research gathered baseline data on GP’s knowledge of and willingness to be involved in DRD prevention, including naloxone administration, prior to the implementation of primary care based delivery.

**Methods:**

Mixed methods were used comprising a quantitative, postal survey and qualitative telephone interviews. A questionnaire was sent to 500 GPs across Scotland. An initial mailing was followed by a reminder. A shortened questionnaire containing seven key questions was posted as a final reminder. Telephone interviews were conducted with 17 GPs covering a range of demographic characteristics and drug user experience.

**Results:**

A response rate of 55% (240/439) was achieved. There was some awareness of the naloxone programme but little involvement (3.3%), 9% currently provided routine overdose prevention, there was little involvement in displaying overdose prevention information (<20%). Knowledge of DRD risk was mixed. There was tentative willingness to be involved in naloxone prescribing with half of respondents willing to provide this to drug users or friends/family. However half were uncertain GP based naloxone provision was essential to reduce DRDs.

Factors enabling naloxone distribution were: evidence of effectiveness, appropriate training, and adding to the local formulary. Interviewees had limited awareness of what naloxone distribution in primary care may involve and considered naloxone supply as a specialist service rather than a core GP role. Wider attitudinal barriers to involvement with this group were expressed.

**Conclusions:**

There was poor awareness of the Scottish National Naloxone Programme in participants. Results indicated GPs did not currently feel sufficiently skilled or knowledgeable to be involved in naloxone provision. Appropriate training was identified as a key requirement.

## Background

In Europe there were an estimated 70,000 drug overdose deaths in 2000-2010 [1]. Scotland has one of the highest rates of Drug Related Deaths (DRDs) in Europe [2]. DRD in Scotland have increased since 1997 and in 2011 there were over 500, one per 10,000 of Scotland’s population. The majority of these deaths involved opiates, usually by injection and concurrent use of benzodiazepines and/or alcohol [3]. Risk factors include a long history of drug use, recent release from prison, psychological stress and homelessness [4]. One intervention that has shown potential effectiveness and cost-effectiveness is take-home naloxone (THN) [5].

Naloxone is a short acting antagonist that temporarily reverses the effects of an opiate overdose, generally administered intra-muscularly. A number of successful projects supplying THN to injecting drug users and their families/friends have been conducted nationally and globally [6-9]. Across Europe just five countries have some form of naloxone distribution (Denmark, Germany, Italy, Romania and the United Kingdom) but this may still be on a small scale [1]. Scotland and Wales are the only countries with national programmes. Naloxone provision through carers and families has been piloted in England through the National Treatment Agency [10]. A full programme across England has not yet been launched. Many other countries are developing pilots e.g. Ireland.

The Scottish National Naloxone Programme (hereafter ‘the naloxone programme’) was launched in 2010 and aims to reduce the high number of DRDs quantified in the most recent National Records [11]. A national programme of training and awareness is provided to support local services delivering THN to those at risk. Each NHS area has a named lead officer, responsible for coordinating delivery at local level. Training aims to raise awareness of overdose risk as well as equipping drug users and family members with Basic Life Support (BLS) skills including naloxone administration [12]. During 2011-12, 2730 naloxone packs were supplied in Scotland; 87% to individuals, 11% to service workers and 2% to friends and family of those at risk [13]. A full THN needs assessment is documented elsewhere [7].

Naloxone is currently largely distributed through specialist drug services in the UK; however the engagement of general practitioners (GPs) is essential to maximise reach. Almost all individuals in the UK are registered with a GP and some drug users may not have access to or wish to use a specialist service. GPs have a particularly important role to play in reducing DRD given that they are the second most common service, after Statutory Addiction Services, with which DRD victims were in contact prior to their death [14]. The potential involvement of GPs in DRD prevention is not a new concept although GPs’ involvement in managing drug users has recently decreased in Scotland [15] which may have impacted on involvement in DRD prevention work.

The “enhanced service” contractual arrangements for substance misuse outline the service level to be provided for substance misuse management by some GPs. Not all GPs manage drug dependence routinely, but all GPs should provide general medical care. Co-morbidity in DRDs is common with over half of cases having a psychiatric condition and almost half having an alcohol problem [5]. Analysis of the causes of premature mortality in a cohort of drug users in Edinburgh found a range of co-morbid conditions including HIV, hepatitis C induced liver disease, kidney failure, respiratory disease and cardio-vascular disease [16]. Thus, drug users may consult their GP even if not in relation to illicit drug problems.

GPs are in an ideal position to either direct patients towards naloxone administration training and supply or to provide it themselves. Additionally, GPs may see family members who could potentially administer naloxone [17]. GP based DRD prevention including naloxone distribution has not previously been documented or evaluated in the international literature. This research aimed to gather baseline data on GP’s current understanding, knowledge and willingness to be involved in DRD prevention, including naloxone administration, prior to considering the implementation of primary care based delivery.

## Methods

The study used mixed methods comprising a quantitative postal survey and qualitative telephone interviews. Both strands ran concurrently and results are presented separately below.

### Quantitative postal survey

A four page questionnaire was developed following familiarisation with the literature and was pre-piloted with a sample of academic GPs. The length of questionnaire was the main issue raised by pilot participants, but it was deemed difficult to further reduce without losing important content.

The questionnaire covered:

•Current GP practice relating to drug misuse generally;

•GP knowledge of DRDs and perception of risk factors [12];

•GP awareness, attitudes and involvement in the naloxone programme including factors that might influence involvement and views on different models of delivery;

•GP training;

•Demographics.

A random sample of 1 in 10 GPs was identified, from a national database listing all GPs in Scotland [18], and stratified by Health Board area to ensure geographical spread. Twenty GPs were used in the pilot sample and 500 for the main distribution. Prior to the main questionnaire mailing, a letter from the Director of Public Health Sciences at NHS Health Scotland was sent to potential participants, endorsing the naloxone programme and encouraging response to the questionnaire which followed approximately one week later. Such pre-notification is considered effective at increasing response rates [19].

The main distribution was mailed in August 2012; a postal reminder was sent two weeks later. Following this, telephone reminder calls were made to managers of practices in which potential participants worked to ensure that the named GP was still in the practice and that the questionnaire had been received. A number of GPs had left, retired, or were on sickness or maternity leave. Since the response remained low, a one-page version of the questionnaire was sent as a final reminder to non-responders, containing only key questions.

### Data analysis

Data were entered into an SPSS V20 database. Simple descriptive statistics (frequencies and distributions) were calculated and cross-tabulations using chi-squared tests were conducted for demographic variables (gender, years of GP experience, specialist training level, geographical location of practice and whether or not participants were currently treating drug misusers) against perceptions of risk factors in relation to DRDs and involvement in and knowledge of the national programme.

### Qualitative interview study

Telephone interviews were conducted to elicit GPs’ views on how to engage the GP community in the naloxone programme. A short topic guide was developed, seeking views on GP involvement, barriers and enablers to involvement as well as experience of naloxone use and drug misuse treatment generally.

A purposive sample was used to cover a range of different levels of experience with patients who have drug problems generally and naloxone specifically. GPs were recruited through the Royal College of General Practitioners (RCGP), Scottish Prison Service, Scottish Primary Care Research Network and NHS Health Scotland; those identified as potential interviewees were contacted by post or e-mail, sent an invitation and information sheet, and an interview time arranged.

Telephone interviews lasted 15-20 minutes and were transcribed verbatim. A basic thematic analysis was undertaken. Emergent themes were identified by one researcher and cross checked by a second. The range of views and experiences under those themes presented. Emergent themes were both topic specific and across topics.

### Ethics

Ethical approval was granted by the College Ethical Review Board of the College of Life Sciences at the University of Aberdeen. Consent for questionnaire participation was implied. Written consent was obtained for interview participants.

## Results

### Quantitative results

A total of 183/439 GPs contacted responded to the long questionnaire. An additional 57 (previous non-responders) returned the short questionnaire giving an overall response rate of 55% for the key questions. Thus the number of respondents was either 183 or 240.

Of the responding GP’s, there was an even gender split, under half (47%) currently treated drug misusers and most had not undertaken any recognised form of specialist training (Table 1); those who had such training also tended to be those treating (77% some training vs 34% no training Pearson Chi-Squared, p < 0.001, data not shown). Only 4% had any specific training on the prevention of DRDs, although the majority expressed an interest in receiving training.

**Table 1:** Questionnaire respondent characteristics

**Male:**			**108/237 (45.6%)**	
**Currently treat drug misusers:**			**113/239 (47.3%)**	
**GP experience: (years)** n = 181	**≤4**	**5-9**	**10-19**	**20+**
	23 (12.6%)	22 (12.0%)	6 2 (33.9%)	76 (41.5%)
**Location** ‡ n = 236	**City Centre:**	**Suburban:**	**Town:**	**Rural:**
	54 (22.9%)	39 (16.5%)	101 (42.8%)	42 (17.8%)
**Specialist training:** † n = 181	**None:**	**Some:**	**More:**	
	127 (70.2%)	29 (16.0%)	25 (13.8%)	

†**None:** none recognised; **Some:** RCGP 1 or local specialist service-run training programmes.

**More:** RCGP 2 or other Credited Post graduate course.

**Town:** 4,000-90,000 inhabitants; **Rural:** <4,000 inhabitants.

While most of the responding GPs were town-based, proportionately more of the city GPs treated drug misuse, 82% compared to 29-49% elsewhere (Pearson Chi-Squared, p < 0.001, data not shown).

43% of respondents did not know how many Drug Related Deaths (DRDs) occur in Scotland and only 12% knew that the actual figure was currently around 500/year. Responses to questions testing knowledge of DRDs are described in Table 2.

**Table 2:** Drug related deaths (DRDs) risk factors

DRD risk factors* (n = 183)	**%**
People who inject	91.3
People recently released from prison	86.9
People who take alcohol with other drugs	86.9
People who take benzodiazepines with other drugs	74.9
Homeless people	74.9
Those newly started on opiate replacement	71.6
People who’ve had additional psychological stress	70.5
People who are under 24 years old †	60.7
People who’ve recently been on a detox programme	59.0
People who’ve used illicit drug for a long time	56.8
**Do you provide overdose prevention:** n = 177	**9.0%**

*Ordered as per proportion, not as in questionnaire.

†Note: not an actual risk factor.

Knowledge of risk factors for DRD is shown in Table 2. While this generally appears high, it is notable that 60% agreed that those aged under 24 years are at higher risk: not a ‘real’ risk factor. While ‘psychological stress’ was ranked lower than the major risk factors it was considered important by 82% of female GPs responding, compared to 60% of their male counterparts (Continuity Correction Chi-Squared, p = 0.002, n = 182) indicating a gender bias.

9% of respondents provided some form of overdose prevention. Just over half had heard of the naloxone programme. However, most did not display information, did not know who their local naloxone lead officer was and were not involved with the national programme (Table 3). Around half knew where to refer drug users for naloxone and almost half were prepared to prescribe/explain naloxone to patients and to patients’ families/friends. Those not prepared to prescribe stated that their main concern was insufficient training/knowledge/experience. Of those prepared to prescribe/explain to patients, 91% would also prescribe/explain to family/friends: a significant trend response (Chi-Squared for Trend, p < 0.001, data not shown) such that their response to one would reliably determine the other.

**Table 3:** Involvement in the Scottish national naloxone programme

**Heard of the national programme? †:**	**n = 240**	**Yes:**	**57.1%**	
**Display/give out infomation on Naloxone?**	n = 181	**Yes:**	18.23%	
**Know local naloxone lead?**	n = 182	**Yes:**	8.2%	
**Involved with national programme?**	n = 181	**Yes:**	3.3%	
**Know where to refer for naloxone?**	n = 181	**Yes:**	55.2%	
**Prepared to prescribe & explain †**	n = 237	**Yes:**	**No:**	**Unsure:**
**naloxone to patients at risk?**		43.9%	22.4%	33.8%
**Prepared to prescribe naloxone & explain to family /friend?**	n = 182	**Yes:**	**No:**	**Unsure:**
		50.5%	22.0%	27.5%

†Included in the final short questionnaire reminder (n = 240).

Training influenced willingness to prescribe and explain naloxone to a patient. While 99 respondents (45%) were willing, when broken down into level of training, significantly more (72%) with the highest training levels were prepared to prescribe naloxone to patients (p = 0.026). There was no significant association between current treatment of drug misusers and willingness to prescribe naloxone to patients or family friends (p = 0.70 and p = 0.85 respectively).

Factors to enable extending the naloxone programme into primary care are described in Table 4. That the programme should be evidence-based was considered very important by most respondents, followed by ‘GP appropriate training’. Conversely, being within the Quality and Outcome Framework (QOF – the system of performance indicators through which GPs are substantially remunerated) was regarded as ‘not important’.

**Table 4:** Importance of factors to extending the naloxone programme (n = 240)

Factors*	**Very**	**Somewhat**	**Not**	**n**
	**important**	**important**	**important**	
Have supporting evidence	**89.7%**	9.5%	0.9%	232
GP appropriate training	**82.8%**	14.2%	3.0%	233
Must be on local formulary	**67.2%**	24.6%	8.2%	232
Practice nurses appropriate training	**52.3%**	30.6%	17.1%	222
GP paid for service	**43.5%**	**39.7%**	16.8%	232
Should be included in the QOF^†^	14.7%	**28.4%**	**56.9%**	225

†QOF: Quality and outcome Framework.

*Ordering with respect to importance.

**Bold%** indicates highest ranked within that factor.

Most GPs who expressed a choice selected the model where GPs only prescribe and others deliver basic life support (BLS) and naloxone training 158/170 (92.9%). Of these, 69% felt someone else should deliver this training (Community Psychiatric Nurse/Specialist Nurse/key worker). There were mixed views on how such an intervention should be delivered i.e. one-to-one or small groups, but more thought take-home naloxone (THN) intervention sessions should be 20-30 minutes rather than brief (<10 minutes).

Once naloxone has been prescribed, debriefing and re-supply was seen by 55% of the GPs to be their responsibility. A further 39% cited ‘others’, mainly outside services such as shared-care clinics, substance misuse services or psychiatric nurses.

Views about the impact of naloxone distribution are shown in Table 5. Half of respondents were uncertain that general practice based distribution was essential to reduce DRDs.

**Table 5:** Attitudes (%) concerning the distribution of naloxone (n = 183)

**Statements**	**n**	**Strongly agree**	**Agree**	**Uncertain**	**Disagree**	**Strongly disagree**
I believe General Practice based distribution of naloxone is essential to reduce drug related deaths.	182	6.6%	19.8%	**49.5%**	14.3%	9.9%
I am concerned that giving injecting drug users naloxone might encourage riskier injecting practices.	183	5.5%	20.9%	24.7%	**37.9%**	11.0%
I am worried that if naloxone is administered by a peer to an injecting drug user they might not phone for an ambulance.	181	9.9%	**40.9%**	28.7%	19.9%	0.6%
I believe the National Naloxone Programme is an important use of NHS resources.	181	5.5%	**40.3%**	**44.8%**	6.6%	2.8%
I feel confident in identifying and addressing overdose risks.	182	2.7%	21.4%	**37.4%**	**34.1%**	4.4%

**Bold**% indicates highest proportion within that Statement.

### Qualitative findings

25 GPs not in the survey sample were approached for an interview: 17 agreed to participate, from a range of locations and experience. One was a face-to-face interview and 16 were conducted by telephone. Most interviewees had some experience with substance misuse patients.

Analysis identified the topic-based themes which mirrored the topics in the interview guide: experience of naloxone, willingness to supply naloxone, opportunistic supply, models of delivery in practice, and barriers.

In addition, there were emergent themes across topics, considered in the discussion. These were: lack of knowledge, ‘typecasting’, ‘off-their-radar’ and negative attitudes.

### Experience of naloxone

There was little experience of using naloxone for opiate overdose. Some had heard of the naloxone programme but were unaware of details. One interviewee had used naloxone while working in a hospital emergency department, another had been involved in a pilot study of THN. Others kept it in their medical bag for emergency use, one has patients involved in the naloxone programme through the shared-care clinic. One practice was previously involved in the nurse-led naloxone programme which stopped when the nurse left.

One practice kept naloxone for emergencies; however, it was not routinely used and never prescribed to take home. Another had patients involved in the naloxone programme, and would participate in the prescribing and speaking to family/peers.

Another interviewee reported that naloxone training was provided by their psychiatric nurse, one occasionally prescribed naloxone on the recommendation of a specialist nurse or to a drug user who had been prescribed it elsewhere and needed re-supply. Prison GPs were familiar with naloxone and its availability would be mentioned at discharge but no specific information provided.

One interviewee familiar with naloxone has discussed it with patients, two of whom expressed an interest. However, he hesitated to prescribe it without having had proper training. Several interviewees had never heard about the naloxone programme.

### Willingness to participate in the naloxone programme

Most interviewees were in favour of participation. A range of motivations were evident with some uncertainty surrounding scope and delivery. Some did not see the need in their own practice as there were few/no currently registered drug users:

‘…we don’t need it, but if we had drug misusers, we would refer them to the drug misuse service.’

Several interviewees commented that this would have to be discussed with other GPs in the practice as sometimes there was a lack of support from colleagues. One in particular had offered to arrange an interview with his partner who opposed treating drug users and naloxone; however the partner was unwilling to talk about his negative views. Another practice provided naloxone training through drug services. Whilst interested, this interviewee did not think that general practice prescribing was required:

‘We already participate to an extent, in that we are supporting patients in accessing the training …I don’t see ourselves prescribing it directly to people, there doesn’t seem to be any particular need…’

Several interviewees were willing to participate if there was a financial benefit:

‘If the financial aspects were favourable......then the motivation for being involved with a heavy time commitment group of patients, might…be attractive.’

Another interviewee supported the programme after experiencing a DRD which may have been preventable had naloxone been available; others would like to receive evidence of clinical benefit before introducing naloxone prescribing. One prison interviewee reported a critical incident that influenced his willingness to prescribe:

‘…a prisoner broke into our drug cupboard room and quite a few people were taking …medications…limited amount of naloxone was stocked and we did run out of naloxone….’

### Opportunistic naloxone

Interviewees were asked how they might deal with a patient who presented for an appointment unrelated to substance misuse, but during the consultation, the GP suspected that they were using drugs and may be at risk of overdose.

Most interviewees did not feel that they have the skills or knowledge to deal with such a situation comfortably and would refer the patient to a substance misuse facility; others would approach the situation, deal with clinical needs, possibly discuss methadone prescribing and then refer to a substance misuse clinic where they can get naloxone:

‘If I knew exactly what to do, I would treat them.....’

‘…may refer them onto more specialist services, or the practice substance misuse clinic.’

One interviewee reported that all patients are routinely asked about drug use at the time of joining the practice; therefore it is unlikely that drug problems would be picked up by coincidence. If a patient has ever used drugs, this will be discussed and if they are regular drug users they will be encouraged to have their own THN supply. Another interviewee reported that if a patient appeared to be a drug user but has come for something else, he will ask outright if they are taking heroin. Others would assess if the patient wants to change their drug using behaviour; if they had no desire to do so, they would give very brief advice. If they indicated that they wanted to change, or get help, they would be referred to drug treatment teams for initial assessment.

### Models of delivery in practice

Interviewees were asked how they could envisage the naloxone programme to be best delivered in general practice. Responses fell into two categories:

### A specialised substance misuse service

Existing models of shared-care programmes and substance misuse services were sufficient and there was no need to change this:

‘The way that it’s done just now…between GP and drug support workers…it would be reasonable to be doing it along the same lines. .’

‘…best mode of delivery is to convey to patients that it is through addiction support…then the kit is provided by the local pharmacy....not delivered through our practice.’

### Non-specialist (GP, nurse or specialist worker)

Several interviewees felt that any clinician or specialist worker could deliver this service, as long as it is the person who has the most contact with the patient. Often this will be a specialist nurse who can more readily assess what is required. Only two interviewees had very strong views that the programme should be GP and not nurse led:

‘…it’s not going to be nurse led…we may take advice of substance misuse nurse, but it’s going to be GP led.’

‘I think from experience of this type of problem, and this type of patient presentation, I can see the benefits of a more specialist role…a GP with a specialist interest…’

Another suggestion was to invite drug users for longer appointments or in a group with families/peers. If naloxone is requested, further information and a referral would be offered.

One interviewee works with a psychiatric nurse to treat drug users but this does not include naloxone provision. He believed if it is to be implemented:

‘…it should be everybody, if you are going to do these things with drug users, it has to be opportunistic, it has to be linked in with other services, and you have to take the opportunity when you get it..’

Similarly another interviewee commented that it should be provided through both general practice and specialist services; otherwise a group of patients may be missed.

### Barriers to naloxone provision

#### Not part of the GP package

Several interviewees mentioned that substance misuse is not part of the core GP contract; overdose prevention information and naloxone training and supply should be part of an enhanced (optional) service contract:

‘…another thing is hoisted on us; we’ve got enough work as it is.’

‘…there are no additional resources to prescribe naloxone, or to talk to patients about naloxone, it’s not part of the enhanced service contract…’

#### Education

It may be difficult to engage GPs to attend educational sessions for several reasons:

‘…fear of looking like an idiot.’

‘…it may ‘take up too much time’

‘GPs may work in an area with low drug misuse and they don’t feel they have the skills.’

Others were concerned about lack of appropriate training and a reluctance to prescribe an injectable drug to a:

‘…chaotic drug user.’

They will need somebody else to administer it which may be difficult as drug users often have a limited and chaotic social circle:

‘…a bit pointless, I’m prescribing it for you, but you are not going to be the person to administer it.’

The need for GPs to be educated and convinced that this is something important to do was noted by others. On-site presentations were suggested to reach the full target group.

#### Safety net

A significant concern was that if drug users have access to naloxone, it may encourage those who were previously frightened to take heroin; it may also tempt current heroin users to increase the amount as they now have perceived safety net:

‘People may think it is safe as long as I’ve got the naloxone there.’

There was concern that when the effect of naloxone wears off and the ambulance has not arrived, a further dose may be needed and still result in a fatal overdose:

‘…I think there would be a concern that…somebody might get a single dose of naloxone and an ambulance not be phoned…it could potentially then be dangerous.’

### Reluctance to treat drug users

Practices not prescribing methadone may consider that engagement with the naloxone programme is too close to becoming involved with drug users. This opinion was voiced by several interviewees; however, one said that even if GPs do not want to treat drug users, they can still provide a possible life saving treatment. Pre-conceived characteristics of drug users were another barrier:

‘…an individual GP prejudice…they may not want to be involved with that type of patient…’

‘Not wanting to treat drug users....it’s got to do with their own attitude....which is pretty shameful, but there you go.’

Not all interviewees were negative: one comment from an interviewee sympathetic to naloxone prescribing noted:

‘…at the end of the day, they are all somebody’s children… it’s very important that GP’s are pro-active and sympathetic in helping…as much as possible.’

## Discussion

The use of mixed methods in this study allowed both national representation and breadth of information alongside in-depth interview data. The survey response rate was initially low, but boosted by the short questionnaire sent as a third reminder. Generating a good response rate from GPs for postal questionnaires is challenging. The final response of 55% compares well with most recent GP survey response rates in the UK [15].

The proportion of the survey respondents currently treating drug users (47%) was comparable to a previous survey in the same population (44%) [15], the level of experience of the naloxone programme was very low with only six individuals being involved.

Knowledge of the number of DRDs in Scotland was surprisingly poor considering that there had been extensive media coverage of the previous year’s DRD figure just before the initial mailing [20]. GPs had low confidence in identifying and addressing overdose risk (24%).

Almost two thirds of respondents had heard of the national programme, mostly from NHS communications. NHS Scotland has thus had some impact in trying to broaden awareness although few knew who their local naloxone lead officer was or who provided naloxone information.

There was uncertainty from survey and interview data about whether general practice was a suitable place for the national programme, although almost half were prepared to prescribe naloxone and explain it to those at risk (or family/friends); a relatively high proportion were ‘unsure’ indicating willingness if certain requirements were fulfilled. It is nevertheless likely that GPs interested in treating problem drug use may have been over-represented in our sample.

Reasons for being ‘unsure’ about prescribing became evident in interviews and centred on a lack of knowledge and the belief that drug treatment is best managed through specialist services; this was evidence of ‘typecasting’ naloxone prescribing as a specialist service.

Half of respondents were uncertain whether general practice based distribution of naloxone was essential to reduce DRDs. This indicates the need for information and ‘evidence’ to convince them that they have a role rather than leaving it to others; this was again echoed in interviews.

Interview data also suggested that practices not treating drug users may consider that naloxone provision is too close to becoming involved with this patient group. Negative attitudes towards drug users indicate underlying attitudinal barriers which could be interpreted as the stigmatisation using current definitions [21]. This is linked to the other emerging themes of typecasting drug misuse treatment as a specialist service. Some GP’s may be keen to ‘offload’ what is perceived as a difficult group to specialist services rather than considering the non-specialist care that is also required; overdose prevention may be part of this.

The existing literature on GPs attitudes to treating drug users is old and our evidence suggests that attitudes may not have changed over time. Training GPs in drug misuse is known to change attitudes [22]; our data support the view that those with more training are more willing to provide naloxone to drug users although causality cannot be assumed. It was noted that the naloxone programme could ‘easily’ be added to the substance misuse enhanced care package.

The main motivating factor in one GP was the experience of a potentially avoidable patient death. This finding concurs with other evidence that experience of a DRD on the caseload of staff does cause a grief-related response [23]. Perhaps this type of experiential evidence could be used to motivate others.

Two models of delivery in general practice were presented in the survey and the preferred one was where GPs only prescribe and others deliver the training. There was uncertainty about whether group or ‘one-to-one’ delivery was preferable, but most believed that the training intervention should be 20-30 minutes. Those preferring a brief intervention type model considered opportunistic delivery preferable. Interviews specifically probed the idea of opportunistic delivery; many perceived this as unrealistic either because they know all their patients or drug users are treated by specialist services.

It was raised in interviews that the intervention could be provided by any health professional in contact with drug users. Other models exist already e.g. pharmacy prescribing in which GPs refer people at risk to the pharmacy. More generally, it was considered that reducing overdose risk should be the priority; otherwise there could be an assumption that someone else is addressing the issue.

### Barriers to GP delivery of the Naloxone Programme

In May 2013, after data collection, a community pack of naloxone was launched (Prenoxad Injection: naloxone 1 mg/1 ml solution) following the granting of a Medicines Licence by the UK Government. This is the world’s first licensed naloxone product for emergency use in non-medical setting by appropriate individuals. It removes any legal/licensing barriers to GP prescribing.

Concerns were expressed that drug users might actually engage in more risky practice by knowing naloxone was available. However, in the survey just a quarter of respondents agreed. The evidence indicates this concern is not realised in practice which should be addressed in any training. It was also suggested that drug users themselves are seen as not aware of their personal risk of a DRD so they might not be receptive to overdose prevention. There is some supportive literature around this concept given that 94.5% of fatalities were considered to be non-deliberate [24]. Rather than a barrier this is further evidence supporting the need for THN.

Stigmatisation of drug users has been considered in detail recently [21]; it has been acknowledged that health professionals can stigmatise drug users and our findings support that. Viewing drug misuse as a health issue rather than a criminal one may result in less stigmatisation [21]. As one interviewee suggested emphasising the lifesaving nature of supplying THN could encourage GPs to provide this intervention to people at risk.

Attitudinal and time barriers clearly exist and are difficult to address. Education and training may overcome some attitudinal barriers but achieving attendance can be challenging and training might need to be taken to practices. There may however also be a need to work with GPs who do not want to treat drug users or do not feel they have the time. Opportunistic naloxone prescribing with a brief intervention to those at risk could be delivered without much commitment to further involvement but only if these GPs were convinced it would be sufficiently safe and could save lives.

### Enablers

Training and the need for evidence supporting THN were considered very important by the majority of questionnaire respondents; most did not think it should be part of the core GP performance payment system, the Quality and Outcomes Framework (QOF). This view may reflect a reluctance to encourage all GPs to do something they may not feel skilled to do or do not want to do.

The need for effective training of clinicians (not just general practitioners) was also recognised in English pilots of a cascading model of training in overdose prevention and THN [25]. Our findings highlighted some essential features for training. There is a strong need for information to have supporting evidence. As noted above the need to emphasise to GPs the key message that naloxone is lifesaving. Training also needs to be delivered sensitively: our interviews indicated that some GPs might feel embarrassed by their lack of knowledge. Furthermore, it should not be assumed that GPs have any level of knowledge of how to administer naloxone. Even those who are used to working with drugs users have rarely been involved in naloxone administration.

### Policy implications

This research raised wider policy issues. Allowing specialised GP services within the GP contract may have exacerbated non-participation of generalist GPs in caring for drug users and lack of familiarity with the concept of preventive naloxone may have provided a justification among some GPs for engaging with what could be interpreted as provision of a new, unfunded, service. Drug users suffer multi-morbidity and die prematurely from a range of health conditions as well as DRDs [5] thus they need general medical care as well as specialist drug treatment. General medical care may be underprovided and this issue requires further assessment. There is evidence of stigmatisation of drug users by GPs in this study and other research; in an analysis of ‘revolving door’ patients i.e. in patients who had been removed from a practice list four or more times, 84% were substance misusers [26].

The negative attitudes of some GPs may underpin the lack of willingness to provide naloxone specifically but may also underpin the lack of willingness to have any involvement with drug users [21]. No recent published research has considered whether GPs stigmatise drug users as a patient group in this way although it is acknowledged that the phenomenon exists [27].

## Conclusions

There was minimal awareness among Scottish GPs of the naloxone programme. Current levels of knowledge and experience of DRD and naloxone use are low, and information needs are high. GPs classify naloxone provision as a specialist service and may therefore assume it is not part of their remit. However, there were signs that some GPs would be willing to be involved if certain enablers were addressed, particularly appropriate evidence-based training. Negative attitudes towards drug users are a barrier to GP care of this patient group. It is possible that changes to the contractual arrangements for GPs may be required to achieve more widespread engagement with the naloxone programme.

## Competing interests

The authors declare that they have no competing interests.

## Authors’ contributions

CM drafted the manuscript, designed the study, obtained ethical approval, coordinated all study related activities and did most of the write up. CPS conducted the qualitative interviews and analysis and drafted the qualitative component of the manuscripts. LA coordinated questionnaire, database development, conducted the statistical analysis and wrote the quantitative results section of the manuscript. AM, ED, SM and PW contributed to the study design and coordination; they also revised the manuscript for important intellectual content. RW advised on specialist GP recruitment and commented on study materials. All authors read and approved the final manuscript.

## Pre-publication history

The pre-publication history for this paper can be accessed here:

http://www.biomedcentral.com/1471-2296/15/12/prepub
